# Low ERK Phosphorylation in Cancer-Associated Fibroblasts Is Associated with Tamoxifen Resistance in Pre-Menopausal Breast Cancer

**DOI:** 10.1371/journal.pone.0045669

**Published:** 2012-09-24

**Authors:** Susann Busch, Lisa Rydén, Olle Stål, Karin Jirström, Göran Landberg

**Affiliations:** 1 Breakthrough Breast Cancer Research Unit, School of Cancer, Enabling Sciences and Technology, University of Manchester, Manchester Academic Health Science Centre, Paterson Institute for Cancer Research, The Christie NHS Foundation Trust, Manchester, United Kingdom; 2 Department of Surgery, Institution of Clinical Sciences, Lund University Hospital, Lund, Sweden; 3 Division of Oncology, Department of Clinical and Experimental Medicine, Linköping University, Linköping, Sweden; 4 Center for Molecular Pathology, Department of Laboratory Medicine, Lund University, Skåne University Hospital Malmö, Malmö, Sweden; 5 Sahlgrenska Cancer Center, Gothenburg University, Gothenburg, Sweden; King Faisal Specialist Hospital & Research Center, Saudi Arabia

## Abstract

**Purpose:**

The aim of this study was to evaluate ERK phosphorylation as a stromal biomarker for breast cancer prognosis and tamoxifen treatment prediction within a randomized tamoxifen trial.

**Patients and Methods:**

Tissue microarrays of two breast cancer cohorts including in total 743 invasive breast cancer samples were analyzed for ERK phosphorylation (pERK) and smooth muscle actin-alpha expression (SMAα) in cancer-associated fibroblasts (CAFs) and links to clinico-pathological data and treatment-predictive values were delineated.

**Results:**

By analyzing a unique randomized tamoxifen trial including breast cancer patients receiving no adjuvant treatment we show for the first time that patients low in ERK phosphorylation in CAFs did not respond to tamoxifen treatment despite having estrogen-receptor alpha (ERα-positive tumors compared to patients with high pERK levels in CAFs (*P* = 0.015, multivariate Cox regression interaction analysis). In both clinical materials we further show a significant association between pERK and SMAα, a characteristic marker for activated fibroblasts. SMAα expression however was not linked to treatment-predictive information but instead had prognostic qualities.

**Conclusion:**

The data suggests that the presence of a subpopulation of CAFs, defined by minimal activated ERK signaling, is linked to an impaired tamoxifen response. Thus, this report illustrates the importance of the stroma for monitoring treatment effects in pre-menopausal breast cancer.

## Introduction

The administration of the anti-estrogen tamoxifen is an adjuvant endocrine therapy for patients with ERα-positive breast cancer. However, many patients do not respond to initial therapy or develop drug resistance and a more patient-tailored therapy approach would be favorable including treatment-predictive markers and alternative treatment options. Therefore, the identification of biomarkers that classify subgroups of breast cancer which will benefit from a particular treatment becomes increasingly relevant [1].

Recently a vast body of literature has emerged demonstrating the importance of the tumor microenvironment (stroma) on tumor progression [2], [3], [4]. Thus, it is evident that exploiting stromal factors will facilitate the discovery of novel biomarkers with prognostic and predictive values [5], [6]. Cancer-associated fibroblasts (CAFs) may be an attractive target due to their abundance in the tumor. CAFs are also referred to as activated fibroblasts or myofibroblasts, and characterized by the presence of mesenchymal markers such as smooth muscle actin-alpha (SMAα) and the absence of epithelial and endothelial markers. However, there is yet no marker unique to CAFs [4] and so far there have been few studies on CAF-specific markers [6].

Activated (phosphorylated) ERK (pERK) has been reported to be a prognostically relevant tumor-specific biomarker in breast cancer and to date, there is a controversy whether activated ERK signaling in tumor cells is associated with better [7] or worse [8], [9] relapse-free survival. Previously, our group reported that ERK phosphorylation in tumor cells of invasive breast cancer was correlated to tamoxifen resistance using three different breast cancer cohorts [10]. However, another group has reported that tamoxifen induces sustained activation of ERK in tumor cells leading to rapid cell death indicating an involvement of ERK signaling in the tamoxifen response of ERα-positive cancer cells [11]. Whether similar effects can be observed *in vivo* and whether basal ERK phosphorylation levels play a role in tamoxifen response however have not been addressed. Moreover the majority of studies focus on ERK signaling within tumor cells neglecting a possible role of the tumor microenvironment on tumor progression or treatment response.

When analyzing ERK phosphorylation in tumor cells breast cancer tissues, we also observed a distinct staining pattern in the stromal compartment. In order to examine the potential prognostic and treatment-predictive values of stromal ERK phosphorylation we therefore analyzed a unique randomized trial including 564 pre-menopausal breast cancer patients randomized to 2 years of tamoxifen or no adjuvant treatment after surgery, as well as a second cohort of 179 pre- and post-menopausal patients and focused on CAFs. The evaluation of the biomarkers was performed according to the REMARK recommendations in order to provide a more transparent and complete report which may improve ascertaining the relevance of the newly found biomarker (Table S5, Figure S4) [12].

## Materials and Methods

### Ethics Statement

The studies were approved by the Ethics Committee at Universities in Linköping and Lund, Sweden (cohort I SBII:2 and cohort II with reference number 447-07). For cohort I, randomization was performed by the Regional Oncological Centers. The Ethics Committees considered that informed consent was not to be required other than by the opt-out method. The data was analyzed anonymously.

### Patients and Tumor Samples

Breast cancer cohort I includes 564 pre-menopausal patients, enrolled in a trial from 1986 to 1991 and randomized to either 2 years of adjuvant tamoxifen treatment (n = 276) or no systemic treatment (n = 288). All patients were followed up for recurrence-free survival. Recurrence was defined as local, regional, or distant recurrence and breast cancer-specific death, whereas contralateral breast cancer was excluded. Each patient underwent surgery (either modified radical mastectomy or breast conserving surgery) followed by radiotherapy and in a small number of cases adjuvant polychemotherapy (less than 2%). The median post-surgery follow-up time without a breast cancer event was 13.9 years. Further details of the trial have been previously described [13], [14]. Breast cancer cohort II includes 179 pre- and post-menopausal patients undergoing endocrine or chemotherapy, diagnosed with primary invasive breast cancer between 2000 and 2002, at the Department of Pathology, Malmö University Hospital. This cohort was designed as a first-line screening cohort for Human Protein Atlas (HPA) antibodies with potential relevance in breast cancer [15]. Median age at diagnosis was 65 years (range 35–97) and median follow-up time 69 months. All patients in this cohort had received treatment following surgery. For detailed description of clinico-pathological features of the tumor samples we refer to previous studies [16], [17]. Representative tumor areas of formalin-fixed and paraffin-embedded tissue material were selected for tissue microarray (TMA) construction. Details regarding TMA assembling and staining procedure have been reported [13].

### Scoring

Scoring of tumor samples was performed independently by a pathologist (G.L.) and a research associate (S.B.) without knowledge of pathological and clinical data. The focus was set on scoring fibroblast adjacent to invasive tumor cells. The scoring accounts for proportion of immunostain-positive fibroblasts. Immunostain scoring for pERK and SMAα was set from no (score = 0), low (score = 1), intermediate (score = 2) to high (score = 3) of stained nucleus and cytoplasm of the fibroblasts.

### Statistical Analyses

Spearman’s rank order correlation coefficient, Pearson’s chi-square test and Mann-Whitney *U* test were performed for evaluation of clinico-pathological and molecular parameters. The Kaplan-Meier method was used to estimate recurrence-free survival and univariate Cox regression was used to compare recurrence-free survival among different treatment groups. Cox proportional hazards regression was used for relative risk estimation in multivariate analysis. Covariates used for Cox regression included tumor grade, tumor size, lymph node status, age, Ki-67 and ERα status. All *P*-values corresponded to two-sided tests and *P*-values less than 0.05 were considered statistically significant.

### Fibroblast Isolation

Primary CAFs were isolated from surgically resected ERα-positive invasive breast carcinomas on the day or following day of surgery. All patients were consented through the Manchester Cancer Research Centre (MCRC) Biobank. Tumour samples were dissected with scalpel and left to digest in DMEM +20% FBS + amino acid solution (Sigma) with 10% Hyaluronidase/Collagenase (Stem Cell Technology) in shaker at 37°C over night. After digestion, cells were filtered through 40 um cell strainer, plated out and cultured until cell number was sufficient for magnetic-activated cell sort (MACS) using anti-CD326 (EpCAM) (Miltenyi) to deplete epithelial cells. Fibroblastic origin was confirmed with immunofluorescence analysis of cytospins using anti-SMAα, anti-Vimentin and anti-Cytokeratin8/18 antibodies (data not shown).

### Western Blot

Isolated primary fibroblasts were plated out in 6 cm^2^ dishes and subjected to serumfree media the following day. Cells were harvested by scraping them off in cell lysis buffer (25 mM HEPES, 5 mM EDTA 30 mM NaPP, 50 mM NaCl, 50 mM NaF, 1% Triton-X, 10% glycerol, pH 7.4) supplemented with protease and phosphatase inhibitor cocktail (Roche). Cells were spun down, the lysate was collected and protein concentration was determined by BCA assay (Pierce). Of each sample 10ug were denatured in 4xLaemmli buffer (250 mM Tris-HCl pH 6.8, 40% glycerol, 8% SDS. 0.01% bromphenol blue, 20% β-mercaptoethanol) and run on 12% SDS-polyacrylamid gel and transferred onto nitrocellulose membrane (Amersham). Membranes were blocked with 5% BSA in TBS-T buffer and subsequently incubated with primary antibody in TBS-T supplemented with 3% BSA and 2% blocking reagent (Roche) in 1∶1000 dilution: rabbit anti-SMAα (Abcam), rabbit anti-phospho-p42/44 MAPK (pERK1/2) (New England Biolabs), rabbit anti-p42/44 MAPK (BD) or rabbit anti-tubulin (New England Biolabs), and after further washing with TBS-T incubated with secondary HRP-linked antibody in 1∶5000 dilution: goat anti-rabbit (Dako). Chemiluminesence was detected using Luminata Forte (Millipore) on X-ray films (Amersham). Membranes were stripped off of antibodies using Re-blot Plus Strong Solution (Millipore) and blocked again prior to reprobing with another primary antibody.

## Results

**Figure 1:**
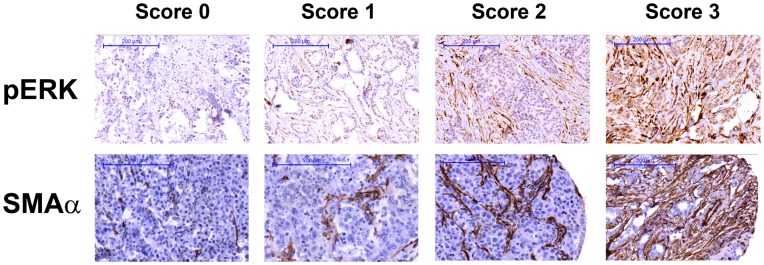
Immunohistochemical staining of tissue microarray sections. Upper panel demonstrating ERK phosphorylation levels (score 0–3) in cancer-associated fibroblasts (CAFs). Lower panel demonstrating SMAα expression (score 0–3) in CAFs. Scale bar represents 200 um. (brown: positive antibody staining, blue/pale pink: haematoxylin/eosin for nucleus and cytoplasm staining, respectively).

ERK phosphorylation (pERK) level in CAFs was divided into four subgroups: negative, low, intermediate and high (score 0–3, respectively) (Figure 1, upper panel) and was then compared to clinico-pathological and molecular parameters (Table 1). In cohort I, there was no significant correlation of pERK in CAFs to major clinico-pathological data such as tumor size, tumor type, Ki-67 status, lymph node status, tumor grade and Her2 but to ERα and progesterone receptor (PR) (*P = *0.006 and *P = *0.004, respectively, Mann-Whitney *U*). However, in cohort II there was a significant inverse relationship to tumor size (*P* = 0.017, Mann-Whitney *U*) but not to ERα or PR (Table S2) maybe due to lower case numbers. Additionally, pERK in CAFs was significantly linked to vascular endothelial growth factor (VEGF) as well as VEGF receptor (VEGFR) expression in the tumor cells (*P = *0.002 and *P*<0.001, respectively, Spearman) (Table 1). Hence, there seems to be a link between ERK phosphorylation in CAFs and PR- and ERα-positive breast cancer with elevated VEGF signaling.

**Table 1:** Prognostic and molecular parameters.

	CAF-pERK				*P*
		
	0	1	2	3	
	n = 152	n = 77	n = 176	n = 20	
**Tumor size**					
≤20	46 (30)	27 (35)	67 (38)	7 (35)	
>20	105 (70)	50 (65)	109 (62)	13 (65)	.184 1
Missing: 1					
**Tumor type**					
Ductal	123 (85)	65 (86)	149 (87)	17 (89)	
Lobular	9 (6)	8 (10)	14 (8)	2 (11)	
Medullary	12 (8)	3 (4)	8 (5)	0 (0)	.548 2
Missing: 15					
**LN status**					
N0	50 (33)	17 (22)	48 (27)	5 (25)	
N+	100 (67)	60 (78)	128 (73)	15 (75)	.243 1
Missing: 0					
**Grade (NHG)**					
I	19 (13)	4 (5)	17 (10)	4 (21)	
II	57 (39)	24 (32)	81 (49)	9 (47)	
III	71 (48)	47 (63)	69 (41)	6 (32)	.152 2
Missing: 17					
**Ki-67**					
≤25%	98 (72)	52 (72)	113 (71)	13 (81)	
>25%	39 (28)	20 (28)	46 (29)	3 (19)	.822 1
Missing: 41					
**ER**α					
≤10%	58 (40)	28 (37)	47 (27)	4 (20)	
>10%	88 (60)	48 (63)	127 (73)	16 (80)	.006 1
Missing: 9					
**PR**					
≤10%	51 (46)	27 (44)	45 (30)	4 (27)	
>10%	61 (54)	34 (56)	106 (70)	11 (73)	.004 1
Missing: 86					
**Her2**					
Negative (≤10%)	81 (62)	42 (61)	78 (50)	10 (55)	
Low	23 (18)	4 (8)	37 (24)	5 (28)	
intermediate	7 (5)	6 (9)	19 (12)	2 (11)	
High	19 (15)	17 (25)	22 (14)	1 (6)	.144 3
Missing: 52					
**CAF-SMA**α					
0	8 (7)	0 (0)	7 (5)	0 (0)	
1	25 (22)	12 (19)	23 (17)	0 (0)	
2	58 (51)	35 (55)	61 (46)	8 (50)	
3	23 (20)	17 (27)	43 (32)	8 (50)	.004 3
Missing: 97					
**VEGF**					
0	26 (18)	5 (7)	11 (7)	1 (5)	
1	50 (35)	17 (23)	52 (32)	5 (25)	
2	45 (31)	29 (39)	55 (34)	11 (55)	
3	23 (16)	23 (31)	46 (28)	3 (15)	.002 3
Missing: 23					
**VEGFR**					
0	51 (34)	14 (19)	17 (10)	1 (5)	
1	59 (40)	27 (37)	64 (37)	3 (16)	
2	28 (19)	22 (30)	57 (33)	6 (32)	
3	10 (7)	10 (14)	35 (20)	9 (47)	<.001 3
Missing: 12					

1 Mann-Whitney *U*,

2 Pearson’s chi-square,

3 Spearman.

Distribution of CAF-pERK staining categorization according to clinico-pathological and molecular characteristics. (CAF: Cancer-associated fibroblasts, percentages in parenthesis).

Fibroblast activation marker SMAα was assessed and classified into four groups: negative, low, intermediate and high (score 0–3, respectively) (Figure 1, lower panel) with the majority of tumors in the intermediate and high subgroups in both cohorts (Table S1 and S2). CAF-pERK levels and SMAα expression were significantly correlated (*P = *0.004, Spearman) (Table 1). In cohort I SMAα was linked to tumor size (*P* = 0.006, Mann-Whitney *U*), lymph node status (*P* = 0.039, Mann-Whitney *U*), Ki-67 (*P* = 0.007, Mann-Whitney *U*) and ERα (*P* = 0.013, Mann-Whitney *U*) (Table S1). However in cohort II only associations to Ki-67 (*P* = 0.014, Mann-Whitney *U*) and additionally tumor type (*P* = 0.007, Pearson chi-square) were observed (Table S2).

When combining negative and low (score 0–1) as well as intermediate and high staining intensity (score 2–3) for both CAF-specific markers and comparing SMAα and pERK, 36.5% of tumors were positive for both markers and 13.7% were double-negative (Figure S1A). 9.1% of tumors only showed high pERK level whereas a large proportion of tumors (40.5%) displayed high SMAα expression with low ERK phosphorylation. A similar distribution of CAF-pERK/SMAα subsets was seen in cohort II (Figure S1A). Thus, it appears that CAFs with high levels of activated ERK signaling represent a subset of SMAα-positive fibroblasts.

**Figure 2:**
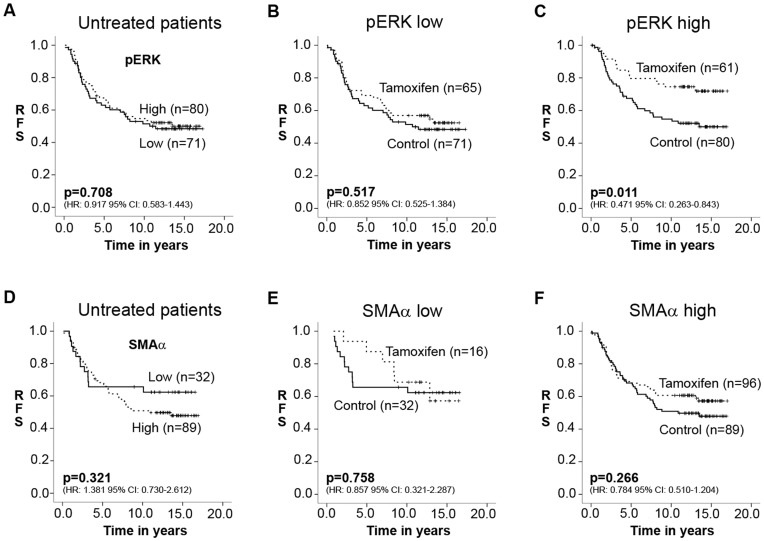
Kaplan-Meier plots. Recurrence-free survival according to CAF-pERK level (A-C) and CAF-SMAα expression (D-F) of patients in cohort I (ERα-positive patients). Plots represent prognostic (A, D) or tamoxifen treatment-predictive information (B, C and E, F) (*P*-value: Univariate Cox regression, HR: Hazard Ratio, CI: Confidence Interval, RFS: Recurrence-Free Survival).

In order to examine the prognostic impact of tumor stroma on survival outcome, we analyzed breast cancer specific recurrences according to pERK or SMAα in CAFs and focused on untreated, ERα-positive tumors in the randomized treatment trial in order to obtain true prognostic information without interference of links to treatment effects. While CAF-pERK was not associated with recurrence-free survival (RFS, plotted as Kaplan-Maier curve) in the untreated control group in cohort I (Figure 2A), higher SMAα levels displayed a tendency towards shorter relapse-free survival (Figure 2D). However, in cohort II multivariate Cox proportional hazard regression analysis revealed an independent prognostic value for CAF-SMAα (Hazard Ratio [HR] = 2.738, 95% confidence interval [CI] = 1.080 to 6.945, *P = *0.034) (Table S3). Patients with tumors exhibiting high levels of SMAα expression in CAFs were subjected to a shorter recurrence-free survival compared to those with tumors of low expression levels (Figure S2A). These data suggest that SMAα-positive CAFs have prognostic features in breast cancer, whereas pERK-positive CAFs are not linked to prognosis.

We next focused on potential tamoxifen treatment-predictive information for the stromal parameters. For comparison of potential association between various types of CAFs and tamoxifen response, we selected patients with ERα-positive breast cancer in cohort I and compared untreated and tamoxifen-treated patients subdivided according to SMAα and pERK. Patients with high ERK phosphorylation in CAFs showed a significant improved recurrence-free survival upon tamoxifen (Hazard Ratio [HR]: 0.471, 95% Confidence Interval [CI]: 0.263 to 0.843, Univariate Cox regression: *P = *0.011) (Figure 2C) similar to the response seen for all ERα-positive breast cancer patients (Hazard Ratio [HR]: 0.620, 95% Confidence Interval [CI]: 0.441 to 0.871, Univariate Cox regression: *P = *0.006) (Figure S2B). However, ERα-positive breast cancer patients with low pERK levels in CAFs had no significant difference in survival outcome between treatment arms (Figure 2B), indicating tamoxifen resistance in this subpopulation of ERα-positive patients. Also tamoxifen-treated patients with low pERK showed a significantly shorter recurrence-free survival in contrast to patients with high pERK in CAFs (Figure 2B & C, respectively). Multivariate Cox regression for CAFs’ pERK marker and treatment interaction analysis revealed a statistically significant difference between the two subgroups defined as low or high pERK (Hazard Ratio [HR] = 2.763, 95% confidence interval [CI] = 1.219 to 6.264, *P = *0.015) (Table 2).

**Table 2:** Multivariate interaction analysis for ERK phosphorylation.

Variable	HR	95% CI	*P*
**Grade (NHG)**			
I-II	1		
III	1.880	1.185–2.983	.007
**Tumor size**			
≤20mm	1		
>20mm	1.261	.813–1.955	.301
**LN status**			
N0	1		
N+	1.229	.763–1.981	.397
**Age**			
Continous (per year)	.962	.929–.996	.030
**Ki67**			
≤25%	1		
>25%	1.225	.710–2.114	.466
**Treatment**			
No tamoxifen	1		
Tamoxifen	.356	.190–.668	.001
**CAF-pERK**			
Low (0–1)	1		
High (2–3)	1.170	.718–1.905	.529
**Interaction**			
pERK x tamoxifen	2.763	1.219–6.264	.015

Recurrence-free survival with Cox proportional hazards regression for relative risk estimation for patients (ERα >10%) in cohort I. (HR: Hazard ratio, CI: Confidence Interval, LN: Lymph node).

In contrast, SMAα expression in CAFs was not associated with a significant difference in tamoxifen response between low and high SMAα as revealed by multivariate Cox regression analysis (Hazard Ratio [HR] = 1.295, 95% confidence interval [CI] = 0.395 to 4.239, *P = *0.669) (Table S4). Furthermore, recurrence-free survival was not shown to be significantly improved upon tamoxifen for either subpopulations (Figure 2E and F).

These results indicate that the subgroup of ERα-positive breast cancer patients comprising CAFs with low pERK level are linked to an impaired tamoxifen response despite having ERα-positive breast cancer cells whereas SMAα level in CAFs is not predictive for tamoxifen response.

We next assessed if a combination of both markers in CAFs was related to tamoxifen treatment effects. Only the double-positive subset exhibited a statistically significant prolonged recurrence-free survival upon tamoxifen treatment (Hazard Ratio [HR]: 0.485, 95% Confidence Interval [CI]: 0.238 to 0.987, Univariate Cox regression: *P = *0.046) (Figure S1C-F). Although case numbers are small for SMAα low subsets, it appears that ERα-positive breast cancer patients only benefit from tamoxifen treatment when the surrounding CAFs exhibit high ERK phosphorylation as well as high SMAα expression.

**Figure 3:**
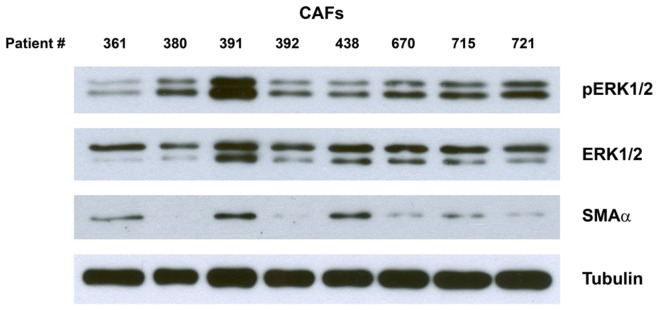
Western blot of primary breast cancer-associated fibroblasts (CAFs). CAFs are derived from patients with ERα-positive breast cancer and have been cultured in serumfree media to allow detection of basal ERK phosphorylation levels (lower band: ERK2 42kDa, upper band: ERK1 44kDa).

To test whether CAFs display different levels of ERK phosphorylation *ex vivo* we obtained breast tumor samples and isolated breast cancer-associated fibroblasts through enzymatic digestion and MACS separation. Only CAFs of tumor samples that were stated ERα-positive by the pathologist were used for ERK phosphorylation analysis by Western blot. CAFs were cultured in serumfree media to examine basal levels of pERK. In Figure 3 eight tumor samples are shown, which demonstrates that primary CAFs can exhibit distinct ERK phosphorylation levels ranging from low (#361) to high (#391). Yet, most samples display intermediate pERK levels. It appears that ERK phosphorylation is independent of SMAα expression, however sample number is small. More strikingly, tumor samples also reveal different expression levels of ERK2 (lower band) whereas ERK1 (upper band) is fairly evenly expressed amongst samples. However, relative phosphorylation levels of ERK1 compared to ERK2 are similar.

## Discussion

This is the first report that demonstrates an impaired tamoxifen response in a subgroup of ERα-positive breast cancer defined by minimal activated ERK signaling (low pERK) in CAFs. We further show that pERK-positive CAFs constitutes a subgroup of tumor-adjacent fibroblasts that are significantly linked to general activation of CAFs as determined by SMAα expression but can further be a separate entity from SMAα positivity. ERα-positive breast tumors with CAFs exhibiting low pERK is predictive for tamoxifen treatment resistance whereas in contrast SMAα-positive CAFs had prognostic qualities illustrating the importance of CAFs in tumor behavior but also supporting the existence of subgroups of CAFs with different prognostic and predictive values. Previously, myofibroblasts have already been described as an extremely heterogeneous and multifunctional cell population exhibiting different phenotypes [18].

The validity of the use of phosphorylation state-specific antibodies in immunohistochemistry, in terms of tumor assessment, is still controversial (reviewed by Mandell et al.) regarding epitope specificity, robustness and reproducibility of immunohistochemical assays. The cut-off from the blood supply when taking tumor samples/biopsies leads to rapid dephosphorylation of phospho-proteins allowing no detection when left unfixed for too long. Yet, many studies have indicated that assessing protein phosphorylation state can add prognostic, predictive and therapeutic monitoring information [19]. In order to internally validate ERK phosphorylation in formalin-fixed paraffin-embedded (FFPE) tissue in this study, we used tumor-specific pERK positivity as determined previously and assumed that differences in fixation procedure and efficiency would inevitably affect both the tumor- and fibroblast-specific levels of pERK [10]. It can further be assumed that the stability of phospho-proteins is similar throughout one tumor specimen as the time until fixation is the most critical part in terms of the strength of the signal upon immunohistochemistry [20]. Analysing the distribution of pERK level in tumor and CAFs revealed that 41.7% of all tumors had low ERK phosphorylation in both cell types (Figure S3A) possibly representing samples with poorly preserved phospho-proteins due to fixation. Excluding patients with low pERK in tumor cells would therefore hypothetically eliminate any poorly fixed and stained tumor samples and any biomarker qualities of pERK in CAFs should be preserved although statistical power may be compromised due to lower case numbers. When selecting for patients displaying high tumor-specific pERK levels thus representing well-preserved tissue in terms of pERK staining, a clear difference in tamoxifen response regarding CAF-specific pERK levels was still observed (Figure S3D and E, respectively). Although we detected only borderline significant improved recurrence-free survival upon tamoxifen in high pERK subgroup (Hazard Ratio [HR] = 0.444, 95% confidence interval [CI] = 0.197 to 1.004, Univariate Cox regression: *P* = 0.051), the low pERK in CAFs displayed no difference in survival between treatment arms (Hazard Ratio [HR] = 1.204, 95% confidence interval [CI] = 0.436 to 3.327, Univariate Cox regression: *P* = 0.720) confirming our finding of an impaired tamoxifen response in patients with low ERK phosphorylation in CAFs.

Hence, we conclude that the presence of low or high level of pERK in CAFs and its relation to tamoxifen response is indeed a valid biomarker. Additionally, ERK phosphorylation as an indicator of active intrinsic signaling may be a better predictor than expression of known clinically relevant markers as it was shown for Akt phosphorylation compared to epidermal growth factor receptor expression in non-small cell lung cancer in relation to gefitinib response [21].

In this study we show that pERK-positive CAFs are significantly but not exclusively linked to SMAα-positive CAFs. The majority of tumor samples further showed high levels of SMAα which is not unexpected as reactive tumor stroma is generally characterized by SMAα expression in the fibroblasts [22]. However, the existence of SMAα-negative or low expressing CAFs imply that the general activation of tumor stroma vary between patients either depending on the nature of the arising cancer or depending on intrinsic signaling. Interestingly, SMAα expression in CAFs was positively correlated with tumor size and the proliferation marker Ki-67 indicating a tumor growth promoting role. Pinto and colleagues could show that co-injection of SMAα-positive CAF and the ERα-positive breast cancer cell line MCF-7 into nude mice increases tumor growth and proliferative activity in MCF-7 cells as well as in the normal adjacent epithelium [23]. ERK phosphorylation in CAFs was not shown to be linked to tumor size or Ki-67 [18].

Strikingly, pERK-positive CAFs were also positively associated with VEGF and VEGFR expression in the tumor cells. VEGF and VEGF signaling is a key player in angiogenesis and metastasis and has been reported to be a marker for poor prognoses [24]. Both estrogen as well as tamoxifen treatment have been associated with an increased VEGF expression but another study could also show that intracellular levels were increased but that secretion of VEGF was inhibited by tamoxifen *in vitro* and *in vivo* [25], [26], [27]. Whether a high level of VEGF before endocrine therapy makes the tumor more susceptible to tamoxifen-induced reduction of VEGF levels is unknown. Also, it is not clear whether high VEGF and VEGFR expression in tumor cells affects cell signaling in the adjacent stroma or whether fibroblasts with high ERK signaling contribute to VEGF/VEGFR expression in the tumor.

Western blot analysis revealed that primary CAFs exhibit different levels of ERK activation. These differences in basal ERK phosphorylation levels indicate an intrinsic capacity of fibroblasts to regulate ERK signaling. Whether the observed variation in ERK phosphorylation is a result of aberrant ERK signaling itself or due to cross-talks with other signaling pathways or a consequence of an altered secretion of autocrine factors needs to be determined. As fibroblasts are generally considered to be genetically stable [28], it is likely that epigenetic events acquired during tumorigenesis prime fibroblasts for a distinct phenotype that may be independent of myofibroblast differentiation accounting for the vast fibroblast heterogeneity within the tumor stroma. We can only assume that isolated primary cells retain their phenotypical profile *ex vivo* in order to support the presented clinical findings. In fact in accordance with the reported clinical data, Western blot confirmed the presence of SMAα negative or low expressing CAFs whereas the correlation between ERK phosphorylation and SMAα expression was less obvious due to small sample size.

We also observed a discrepancy in expression levels between ERK1 and ERK2 in primary CAFs. Generally pERK1 and pERK2 levels are taken together to represent total ERK activity but it has been suggested that both ERK kinases have distinct cellular functions [29]. Several studies have shown that ERK2 knockout mice die early in development whereas ERK1-deficient mice are viable with minor defects [30], [31]. Furthermore, mouse embryo fibroblasts (MEFs) isolated from knockout mice showed that ERK1-deficient MEFs proliferated faster and exhibited an elevated level of ERK2 activation compared to control cells. The same effect of an increased proliferation rate and enhanced ERK2 activity was seen knocking down ERK1 using lentiviral shRNA while ERK2 knockdown cells proliferated poorly, suggesting that ERK2 mediates proliferative signals whereas ERK1 may have inhibitory effects [32]. However, whether regulation of ERK2 expression and activation affects CAF proliferation and thereby altering tumor-stromal interaction is entirely speculative at the present.

So far, it is unclear how fibroblasts with a diminished or elevated basal ERK phosphorylation level evolve within the tumor stroma. ERK-related studies have focused mainly on epithelial/tumor cells and little is known about the role and regulation of ERK in fibroblasts. However, in a report studying dermal wound healing ERK signaling in fibroblasts seemed mainly to be involved in increased proliferation [33]. It is noteworthy that fibroblast differentiation through TGFβ1 is accompanied with an activation of ERK signaling [34], [35]. Recently it has been demonstrated that estrogen can induce gene expression and increase migration in mammary CAFs by an ERα-independent pathway through GPR30-mediated transactivation of EGFR leading to activation of ERK [36]. The question whether this hormonal regulation also occurs in normal stroma was nevertheless not addressed. In summary, ERK activation in fibroblasts may be part of the differentiation process as the tumor progresses, through secretion of tumor-derived factors such as TGFβ and PDGF or via hormonal regulation ie. estrogen. Consequentially, fibroblast proliferation, migration and gene expression of differentiated CAFs will be distinct compared to the physiological role of stromal tissue. Notably, as activation of the ERK-MAPK pathway has been linked with key events in cell transformation and is therefore attractive for therapeutic targeting, the impact of MAPK inhibitors on subsets of CAFs might in fact influence treatment-predictive information in breast cancer [37], [38].

The effect of tamoxifen on fibroblasts is also matter of speculation. Either a distinct subtype of stroma is a mere indicator for tumors susceptible to tamoxifen treatment or tamoxifen directly induces changes in fibroblasts which mediate signals to the tumor cells resulting in an altered tamoxifen response. This illustrates the complexity of the dynamic and reciprocal nature of the tumor-stromal interaction. Hattar and colleagues have shown that tamoxifen induces changes in the rat mammary stroma creating a microenvironment that is inhibitory to tumor cell progression [39]. Furthermore, co-culture experiments revealed that tamoxifen sensitivity or resistance of breast cancer cell lines was mediated through fibroblasts isolated from breast tumors [40]. These studies suggest that tamoxifen affects mammary stroma directly and causes remodeling of the tumor microenvironment, defining the cell response of the tumor upon tamoxifen treatment. Evaluation of additional patient cohorts and further *in vitro* and *in vivo* experimental models are required to confirm whether fibroblasts with diminished ERK signaling confer a distinct tamoxifen outcome compared to normal or differentiated fibroblasts with higher pERK, supporting the idea of a fibroblast-mediated tamoxifen response.

In the past decade the significance of stromal gene signatures have been reported by numerous studies [41], [42], [43], [44], [45], [46]. However, using different data sets and data analysis approaches and according to the question addressed, this led to various stromal response patterns which could be linked to clinical phenotype or tumor progression (therapy resistance, survival outcome). These prognostic and predictive implications highlight firstly the importance of the stromal compartment on tumor progression and secondly demonstrate the heterogeneity of tumor stroma regarding tumor characteristics. To date, no stromal gene expression profile predicting tamoxifen response is available to evaluate whether ERK pathway or target genes are upregulated in tamoxifen responsive tumors. For future clinical routine analyses, ideally a limited amount of treatment-predictive stromal and epithelial markers should be defined. Consequent analysis of those markers using immunohistochemical platforms widely available in hospitals may avoid time-consuming and expensive microdissection and more complex expression array profiling. Additional studies nevertheless have to prove how useful pERK is as a marker for tamoxifen resistance in pre-menopausal breast cancer as well as identifying alternative and potentially more stable markers for activation of ERK that can be useful in clinical routine analyses.

In summary, our study supports the idea that in addition to conventional tumor markers also stromal biomarkers possess treatment-predictive information and could therefore be highly valuable in identifying patient subgroups benefiting from endocrine treatment. Moreover ongoing controversies whether certain tumor markers such as ERK phosphorylation are markers for good or poor prognosis may be a consequence of overlooking stromal effects.

## Supporting Information

Figure S1 **Relationship of pERK and SMAα expression.** (A, B) Venn diagrams of CAF-pERK/SMAα proportions in cohort I (A) and in cohort II (B). (C-F) Recurrence-free survival (Kaplan-Meier plots) of CAF-pERK/SMAα subsets in cohort I (ERα-positive patients). (P-value: Univariate Cox regression, HR: Hazard Ratio, CI: Confidence Interval, RFS: Recurrence-Free Survival, CAF: Cancer-associated fibroblast).
(PDF)

Figure S2 **Kaplan-Meier plots.** (A) Recurrence-free survival of all ERα-positive patients in cohort II with regard to CAF-SMAα. (B) Recurrence-free survival of ERα-positive patients according to treatment arms in cohort I. (P-value: Univariate Cox regression, HR: Hazard Ratio, CI: Confidence Interval, RFS: Recurrence-Free Survival, CAF: Cancer-associated fibroblast).
(PDF)

Figure S3 **Relationship of tumor- and CAF-specific pERK.** (A) Venn diagram of tumor- and CAF-pERK proportions in cohort I (total of 415 patients). (B–E) Recurrence-free survival (Kaplan-Meier plots) of ERα-positive patients in cohort I exhibiting low tumor-pERK (B, C) and high tumor-pERK (D, E) in regard to CAF-pERK. (P-value: Univariate Cox regression, HR: Hazard Ratio, CI: Confidence Interval, RFS: Recurrence-Free Survival, CAF: Cancer-associated fibroblast).
(PDF)

Figure S4 **Study design.** Flow diagram of selected patients in cohort I. Event is defined as incidence of recurrence (FFPE: Formalin-fixed paraffin-embedded, TMA: tissue microarray).
(PDF)

Table S1 **Prognostic and molecular parameters.** Distribution of CAF-SMAα staining categorization according to clinico-pathological and molecular characteristics in cohort I. (CAF: Cancer-associated fibroblasts, percentages in parenthesis).
(PDF)

Table S2 **Prognostic and molecular parameters of cohort II.** Distribution of CAF-pERK and CAF-SMAα staining categorization according to clinico-pathological and molecular parameters in cohort II. (LN: Lymph node, CAF: Cancer-associated fibroblasts, percentages in parenthesis).
(PDF)

Table S3 **Multivariate analysis for SMAα in cohort II.** Recurrence-free survival with Cox proportional hazards regression for relative risk estimation for ERα-positive patients in cohort II. (HR: Hazard ratio, CI: Confidence Interval, CAF: Cancer-associated fibroblasts, LN: Lymph node).
(PDF)

Table S4 **Multivariate interaction analysis for SMAα.** Recurrence-free survival with Cox proportional hazards regression for relative risk estimation for patients (ERα >10%) in cohort I (HR: Hazard ratio, CI: Confidence interval, CAF: Cancer-associated fibroblasts, LN: Lymph node).
(PDF)

Table S5 **Specifications of REMARK recommendations.**
(PDF)
