# Supplementary figures and images for: Low ERK Phosphorylation in Cancer-Associated Fibroblasts Is Associated with Tamoxifen Resistance in Pre-Menopausal Breast Cancer
Source: PLoS One. 2012 Sep 24;7(9):e45669. doi: 10.1371/journal.pone.0045669 (PMC3454403; doi:10.1371/journal.pone.0045669)

Figure S1.

**A**

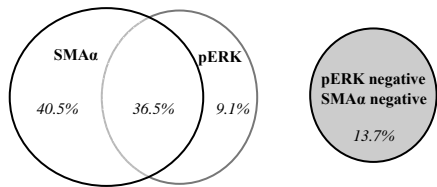

**B**

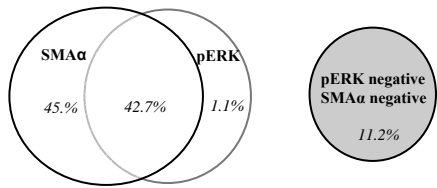

**C**

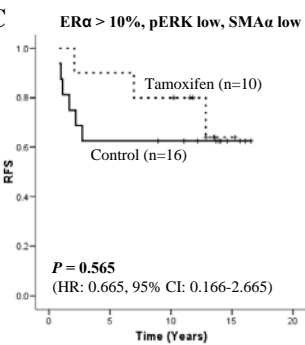

**D**

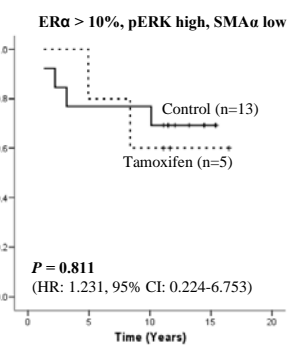

**E**

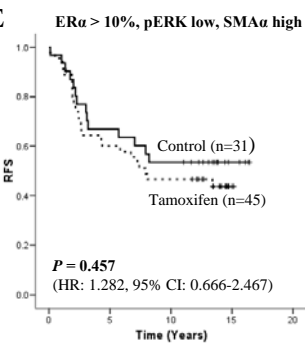

**F**

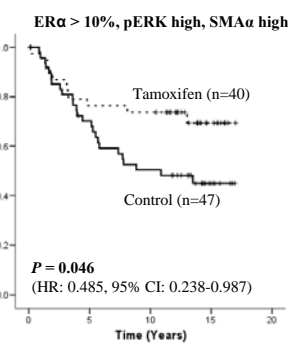

Supplement: Figure S1 — Relationship of pERK and SMAα expression. (A, B) Venn diagrams of CAF-pERK/SMAα proportions in cohort I (A) and in cohort II (B). (C-F) Recurrence-free survival (Kaplan-Meier plots) of CAF-pERK/SMAα subsets in cohort I (ERα-positive patients). (P-value: Univariate Cox regression, HR: Hazard Ratio, CI: Confidence Interval, RFS: Recurrence-Free Survival, CAF: Cancer-associated fibroblast). (PDF) [file pone.0045669.s001.pdf]

Figure S2.

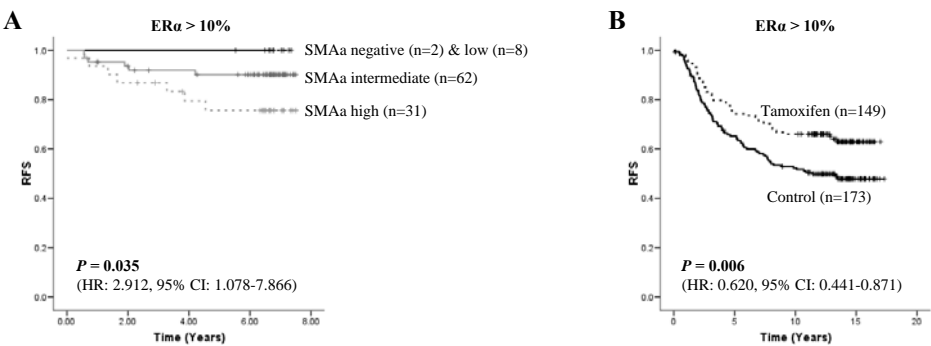

Supplement: Figure S2 — Kaplan-Meier plots. (A) Recurrence-free survival of all ERα-positive patients in cohort II with regard to CAF-SMAα. (B) Recurrence-free survival of ERα-positive patients according to treatment arms in cohort I. (P-value: Univariate Cox regression, HR: Hazard Ratio, CI: Confidence Interval, RFS: Recurrence-Free Survival, CAF: Cancer-associated fibroblast). (PDF) [file pone.0045669.s002.pdf]

Figure S3.

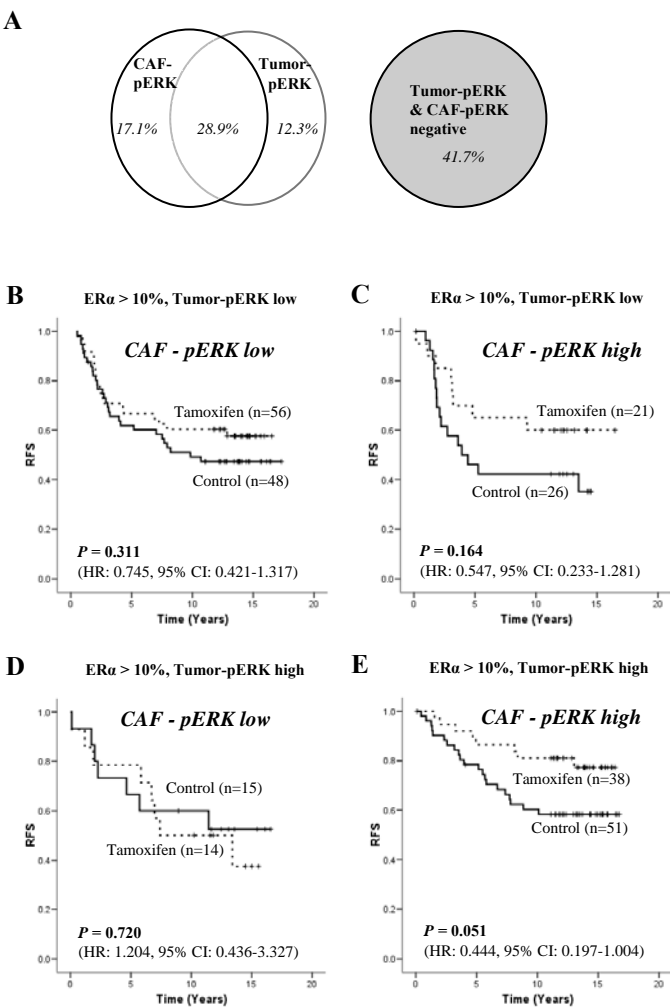

Supplement: Figure S3 — Relationship of tumor- and CAF-specific pERK. (A) Venn diagram of tumor- and CAF-pERK proportions in cohort I (total of 415 patients). (B–E) Recurrence-free survival (Kaplan-Meier plots) of ERα-positive patients in cohort I exhibiting low tumor-pERK (B, C) and high tumor-pERK (D, E) in regard to CAF-pERK. (P-value: Univariate Cox regression, HR: Hazard Ratio, CI: Confidence Interval, RFS: Recurrence-Free Survival, CAF: Cancer-associated fibroblast). (PDF) [file pone.0045669.s003.pdf]

Figure S4.

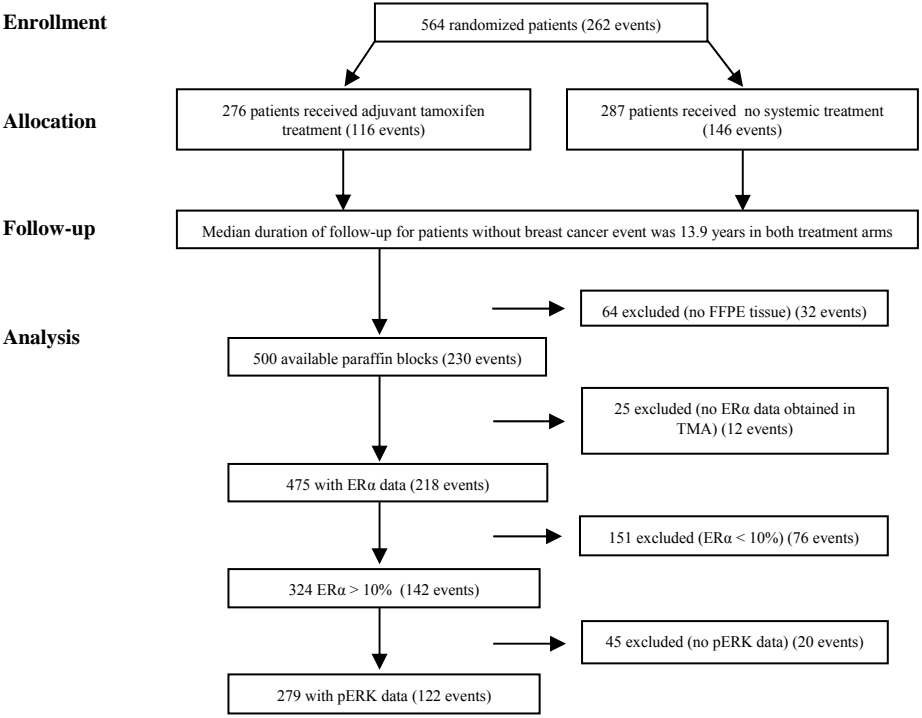

Supplement: Figure S4 — Study design. Flow diagram of selected patients in cohort I. Event is defined as incidence of recurrence (FFPE: Formalin-fixed paraffin-embedded, TMA: tissue microarray). (PDF) [file pone.0045669.s004.pdf]
