# Supplementary material for: Low ERK Phosphorylation in Cancer-Associated Fibroblasts Is Associated with Tamoxifen Resistance in Pre-Menopausal Breast Cancer
Source: PLoS One. 2012 Sep 24;7(9):e45669. doi: 10.1371/journal.pone.0045669 (PMC3454403; doi:10.1371/journal.pone.0045669)
Supplement: Table S1 — Prognostic and molecular parameters. Distribution of CAF-SMAα staining categorization according to clinico-pathological and molecular characteristics in cohort I. (CAF: Cancer-associated fibroblasts, percentages in parenthesis). (PDF) [file pone.0045669.s005.pdf]

Table S1. Prognostic and molecular parameters.

	CAF-SMAα				*P*
	**0**<br>n=19	**1**<br>n=65	**2**<br>n=177	**3**<br>n=106	
**Tumor size**					
≤ 20	4 (21)	20 (31)	51 (29)	49 (46)	
> 20	15 (79)	44 (69)	126 (71)	57 (54)	.006[1]
Missing: 1					
**Tumor type**					
Ductal	14 (78)	51 (79)	149 (86)	94 (92)	
Lobular	3 (17)	7 (11)	11 (6)	5 (5)	
Medullary	1 (6)	7 (11)	13 (8)	3 (3)	.160 [2]
Missing: 9					
**LN status**					
N0	8 (42)	21 (32)	55 (31)	23 (22)	
N+	11 (58)	44 (68)	121 (69)	83 (78)	.039 [1]
Missing: 1					
**Grade (NHG)**					
I	0 (0)	11 (17)	6 (35)	15 (15)	
II	9 (53)	18 (28)	75 (44)	46 (45)	
III	8 (47)	35 (55)	91 (53)	42 (41)	.214 [2]
Missing: 11					
**Ki-67**					
≤ 25%	10 (59)	39 (68)	106 (66)	80 (83)	
> 25%	7 (41)	18 (32)	54 (34)	16 (17)	.007 [1]
Missing: 37					
**ERα positive**					
≤ 10%	8 (44)	23 (37)	64 (38)	24 (23)	
> 10%	10 (56)	39 (63)	104 (62)	81 (77)	.013 [1]
Missing: 14					
**PR positive**					
≤10%	7 (50)	16 (36)	64 (42)	24 (27)	
> 10%	7 (50)	28 (64)	89 (58)	64 (73)	.060 [1]
Missing: 68					
**Her2**					
Negative (≤ 10%)	10 (59)	35 (61)	85 (54)	59 (63)	
Low	4 (24)	12 (21)	23 (15)	17 (18)	
intermediate	1 (59)	3 (5)	17 (11)	6 (64)	
High	2 (12)	7 (12)	33 (21)	12 (13)	.728 [3]
Missing: 41					
**VEGF**					
0	4 (29)	7 (11)	18 (11)	8 (8)	
1	2 (14)	24 (39)	46 (28)	37 (35)	
2	4 (29)	18 (30)	58 (35)	39 (37)	
3	4 (29)	12 (20)	44 (27)	21 (20)	.590 [3]
Missing: 21					
**VEGFR**					
0	3 (17)	14 (23)	30 (18)	26 (25)	
1	9 (50)	23 (38)	65 (38)	29 (28)	
2	5 (3)	18 (30)	42 (25)	36 (35)	
3	1 (6)	6 (33)	33 (19)	11 (11)	.768 [3]
Missing: 16					

[1] Mann-Whitney *U*, [2] Pearson's chi-square, [3] Spearman

Distribution of CAF-SMAα staining categorization according to clinico-pathological and molecular characteristics in cohort I. (CAF: Cancer-associated fibroblasts, percentages in parenthesis)
