# Supplementary material for: Low ERK Phosphorylation in Cancer-Associated Fibroblasts Is Associated with Tamoxifen Resistance in Pre-Menopausal Breast Cancer
Source: PLoS One. 2012 Sep 24;7(9):e45669. doi: 10.1371/journal.pone.0045669 (PMC3454403; doi:10.1371/journal.pone.0045669)
Supplement: Table S2 — Prognostic and molecular parameters of cohort II. Distribution of CAF-pERK and CAF-SMAα staining categorization according to clinico-pathological and molecular parameters in cohort II. (LN: Lymph node, CAF: Cancer-associated fibroblasts, percentages in parenthesis). (PDF) [file pone.0045669.s006.pdf]

Table S2. Prognostic and molecular parameters of cohort II.

	CAF-pERK				*P*	CAF-SMAα				*P*
	0	1	2	3		0	1	2	3	
	n=29	n=32	n=36	n=11		n=2	n=9	n=71	n=38	
**Tumor size**										
≤ 20	11 (38)	14 (45)	20 (56)	9 (82)		0 (0)	1 (11)	39 (55)	18 (49)	
> 20	18 (62)	17 (55)	16 (44)	2 (18)	.017 [1]	2 (100)	8 (89)	32 (45)	19 (51)	.277 [1]
Missing: 1										
**Tumor type**										
Ductal	19 (70)	22 (71)	24 (73)	5 (56)		0 (0)	5 (56)	51 (78)	31 (86)	
Lobular	6 (22)	9 (29)	8 (24)	4 (44)		2 (100)	3 (33)	14 (22)	3 (8)	
Medullary	2 (7)	0 (0)	1 (3)	0 (0)	.591 [2]	0 (0)	1 (11)	0 (0)	2 (6)	.007 [2]
Missing: 8										
**LN status**										
N0	20 (71)	15 (52)	17 (59)	7 (70)		2 (100)	6 (67)	34 (55)	18 (55)	
N+	8 (29)	14 (48)	12 (41)	3 (30)	.166 [1]	0 (0)	3 (33)	28 (45)	15 (45)	.332 [1]
Missing: 12										
**Grade (NHG)**										
I	4 (13)	0 (0)	5 (14)	3 (27)		0 (0)	0 (0)	12 (17)	5 (14)	
II	14 (48)	17 (55)	18 (50)	6 (55)		1 (50)	6 (67)	31 (44)	13 (35)	
III	11 (38)	14 (45)	13 (36)	2 (18)	.421 [2]	1 (50)	3 (33)	28 (39)	19 (51)	.312 [2]
Missing: 1										
**Ki-67**										
Low	1 (4)	0 (0)	0 (0)	3 (30)		0 (0)	0 (0)	1 (2)	5 (14)	
Moderate + High	23 (96)	26(100)	32(100)	7 (70)	.075 [1]	2 (100)	9 (100)	55 (98)	31 (86)	.014 [1]
Missing: 16 (pERK) 17 (SMAα)										
**ERα positive**										
≤ 10%	6 (21)	3 (10)	4 (11)	0 (0)		0 (0)	1 (11)	9 (13)	6 (16)	
> 10%	23 (79)	28 (90)	32 (89)	11(100)	.105 [1]	2 (100)	8 (89)	62 (87)	31 (84)	.496 [1]
Missing: 1										
**PR positive**										
≤ 10%	10 (34)	10 (32)	9 (25)	1 (9)		1 (50)	3 (33)	23 (32)	13 (35)	
> 10%	19 (66)	21 (68)	27 (75)	10 (91)	.126 [1]	1 (50)	6 (67)	48 (68)	24 (65)	.915 [1]
Missing: 1										
**Her2**										
Negative (≤ 10%)	21 (75)	18 (60)	20 (57)	7 (64)		2 (100)	6 (67)	43 (64)	19 (53)	
low	4 (14)	7 (23)	9 (26)	3 (27)		0 (0)	1 (11)	15 (22)	11 (31)	
intermediate	2 (7)	2 (7)	4 (11)	0 (0)		0 (0)	2 (22)	5 (7)	2 (6)	
high	1 (4)	3 (10)	2 (6)	1 (9)	.295 [3]	0 (0)	0 (0)	4 (6)	4 (11)	.193 [3]
Missing: 4 (pERK) 6 (SMAα)										
**CAF-SMAα**										
0	1 (4)	0 (0)	1 (4)	0 (0)						
1	6 (26)	3 (11)	0 (0)	0 (0)						
2	14 (61)	17 (63)	14 (50)	6 (55)						
3	2 (9)	7 (26)	13 (46)	5 (45)	<.001 [3]					
Missing: 19										

[1] Mann-Whitney *U*, [2] Pearson's chi-square, [3] Spearman

Distribution of CAF-pERK and CAF-SMAα staining categorization according to clinico-pathological and molecular parameters in cohort II. (LN: Lymph node, CAF: Cancer-associated fibroblasts, percentages in parenthesis)
