# Supplementary material for: Low ERK Phosphorylation in Cancer-Associated Fibroblasts Is Associated with Tamoxifen Resistance in Pre-Menopausal Breast Cancer
Source: PLoS One. 2012 Sep 24;7(9):e45669. doi: 10.1371/journal.pone.0045669 (PMC3454403; doi:10.1371/journal.pone.0045669)
Supplement: Table S3 — Multivariate analysis for SMAα in cohort II. Recurrence-free survival with Cox proportional hazards regression for relative risk estimation for ERα-positive patients in cohort II. (HR: Hazard ratio, CI: Confidence Interval, CAF: Cancer-associated fibroblasts, LN: Lymph node). (PDF) [file pone.0045669.s007.pdf]

Table S3. Multivariate analysis for SMAα in cohort II.

Variable	HR	95% CI	*P*
**Grade (NHG)**			
I-II	1		
III	2.414	.630-9.246	.198
**Tumor size**			
≤ 20mm	1		
> 20mm	2.658	.544-12.990	.227
**LN status**			
N0	1		
N+	4.629	1.258-17.036	.021
**Age**			
Continous (per year)	1.030	.998-1.062	.067
**Ki67**			
≤ 25%	1		
> 25%	.376	.030-4.728	.449
**CAF-SMAα**			
0			
1			
2			
3	2.738	1.080-6.945	.034

Recurrence-free survival with Cox proportional hazards regression for relative risk estimation for ERα-positive patients in cohort II. (HR: Hazard ratio, CI: Confidence Interval, CAF: Cancer-associated fibroblasts, LN: Lymph node)
