# Supplementary material for: Low ERK Phosphorylation in Cancer-Associated Fibroblasts Is Associated with Tamoxifen Resistance in Pre-Menopausal Breast Cancer
Source: PLoS One. 2012 Sep 24;7(9):e45669. doi: 10.1371/journal.pone.0045669 (PMC3454403; doi:10.1371/journal.pone.0045669)
Supplement: Table S4 — Multivariate interaction analysis for SMAα. Recurrence-free survival with Cox proportional hazards regression for relative risk estimation for patients (ERα >10%) in cohort I (HR: Hazard ratio, CI: Confidence interval, CAF: Cancer-associated fibroblasts, LN: Lymph node). (PDF) [file pone.0045669.s008.pdf]

Table S4. Multivariate interaction analysis for SMAα.

Variable	HR	95% CI	*P*
**Grade (NHG)**			
I-II	1		
III	1.630	.994-2.674	.053
**Tumor size**			
≤ 20mm	1		
> 20mm	1.171	.719-1.905	.526
**LN status**			
N0	1		
N+	1.579	.908-2.745	.105
**Age**			
Continous (per year)	.963	.922-1.006	.092
**Ki67**			
≤ 25%	1		
> 25%	1.302	.743-2.281	.356
**Treatment**			
No tamoxifen	1		
Tamoxifen	.730	.459-1.162	.184
**CAF-SMAα**			
Low (0-1)	1		
High (2-3)	1.604	.793-3.244	.189
**Interaction**			
SMAα x tamoxifen	1.295	.395-4.239	.669

Recurrence-free survival with Cox proportional hazards regression for relative risk estimation for patients (ERα > 10%) in cohort I (HR: Hazard ratio, CI: Confidence interval, CAF: Cancer-associated fibroblasts, LN: Lymph node)
