# Supplementary material for: Low ERK Phosphorylation in Cancer-Associated Fibroblasts Is Associated with Tamoxifen Resistance in Pre-Menopausal Breast Cancer
Source: PLoS One. 2012 Sep 24;7(9):e45669. doi: 10.1371/journal.pone.0045669 (PMC3454403; doi:10.1371/journal.pone.0045669)
Supplement: Table S5 — Specifications of REMARK recommendations. (PDF) [file pone.0045669.s009.pdf]

## Table S5. Specifications of REMARK recommendations.

| Introduction | |
|---|---|
| Marker | Antibody for phosphorylation of extracellular signal-regulated kinase ERK1/2 (Phospho-p44/42 MAPK) (Cell Signaling Technology #9101) detects endogenous levels of p44 and p42 MAP Kinase (ERK1 and ERK2) when phosphorylated either individually or dually at Thr202 and Tyr204 of ERK1 (Thr185 and Tyr187 of ERK2) assessed by immunohistochemistry. |
| Objectives | To evaluate the predictive capacity of ERK phosphorylation in tumour-associated fibroblasts with regard to tamoxifen response in premenopausal breast cancer |
| Hypothesis | An earlier study has shown that ERK phosphorylation in tumour cells is linked to tamoxifen response [1]. A distinct level of ERK phosphorylation was observed in the surrounding fibroblasts. We hypothesise that ERK phosphorylation in fibroblasts possess treatment predictive information. |

| Methods (1) | Patients |
|---|---|
| Characteristics | From 1986 until 1991, 564 premenopausal stage II patients from two centres in Sweden were enrolled in a randomized trial (SBII:2a) and randomly assigned to either 2 years of adjuvant tamoxifen (n=276) or no systemic treatment (control) (n=288).[2] Premenopausal patients or patients younger than 50 years with stage II (pT2 N0 M0, pT1 N1 M0. or pT2 N1 M0) invasive breast cancer were included. Patients were considered premenopausal until 1 year after the last menstruation. |
| Inclusion criteria | For this translational study, patients with an ERα in more than 10% of the invasive tumour cells were included. |
| Exclusion criteria | See Figure S5. |
| Treatment | Patients were randomly assigned to either 2 years of adjuvant tamoxifen or no systemic treatment (control). All patients received radical surgery in the form of a modified radical mastectomy or breast-conserving surgery with axillary lymph node dissection (levels one and two). After breast-conserving surgery, radiotherapy (50 Gy) was given to the breast, and in patients with axillary lymph node metastases, locoregional radiotherapy was delivered. Study centre 1 used a daily dosage of 40 mg of tamoxifen, whereas study centre 2 used a daily dosage of 20 mg of tamoxifen. In postmenopausal patients, the results of tamoxifen treatment 20 mg or 40 mg daily have been reported to be similar (22). Adjuvant polychemotherapy with cyclophosphamide, methotrexate, and fluorouracil (CMF) or goserelin was administered in nine patients (<2%). |

| Methods (2) | Specimen characteristics |
|---|---|
| Material used | Formalin-fixed paraffin-embedded breast tumour tissue of the primary tumour. Two 600-μm cores were used for construction of tissue microarrays (TMAs). |
| Control samples | Random samples of invasive breast cancer included in TMAs were stained with different dilutions of the antibody and different protocols for antigen retrieval before staining the actual trial TMAs. The protocol that resulted in specific nuclear staining in tumour cells but also resulted in samples with negative nuclei was used for staining the trial TMAs. |
| Preservation/Storage | Formalin fixation and paraffin embedding. Storage at room temperature. |

| Methods (3) | Assay methods |
|---|---|
| Assay used | Polyclonal antibody (Cell signaling, product 9101) reacting with ERK only when phosphorylated at either individually or dually at Thr202 and Tyr204 of ERK1 (Thr185 and Tyr187 of ERK2). Produced by immunizing animals with a synthetic phospho-peptide (KLH-coupled) corresponding to residues surrounding Thr202/Tyr204 of human p44 MAP kinase. Antibodies are purified by protein A and peptide affinity chromatography. |

| | |
|---|---|
| | Antibodies are purified by protein A and peptide affinity chromatography. Antibody specificity was validated previously using Western blot in parallel to immunohistochemistry of formalin-fixed paraffin-embedded cell lines [1]. |
| Protocol | Construction of the TMAs has been described previously [3]. Sections (4 μm) were mounted onto slides, deparaffinized with xylene, and rehydrated in graded ethanol series. The slides were then processed in a Pascal pressure chamber (S2800, Dako, Glostrup, Denmark) for 1 min in target retrieval solution (Dako), pH 9.9, and stained with a mouse monoclonal antibody against pERK (1:100 dilution) in a Techmate 500 automated immunostainer by use of Envision software (Dako). pERK was detected with diaminobenzidine. |
| Reproducibility | Scoring the pERK staining by two observers (SB and GL) resulted in a kappa of 0.902. |
| Quantification | pERK staining was interpreted via the Allred scoring system [4]. Intensity score: 0 = none; 1 = weak; 2 = intermediate; 3 = strong. |
| Blinding | Observers (SB and GL) that scored the TMAs stained with the pERK antibody had no knowledge of both the recurrence-free survival and the treatment arm at the time of scoring. |

| **Methods (4)** | **Study design** |
|---|---|
| Case selection | A randomized controlled trial. The translational study presented here was performed retrospectively. Stratification for tumour size or lymph node status was not included in the protocol. From January 1, 1986, to September 30, 1991, patients were enrolled in the study. The median duration of follow-up for patients without a breast cancer event was 13.9 years (95% confidence interval [CI] = 13.6 to 14.3), and median follow-up time was the same in the two treatment arms. Patient records were re-evaluated from 2002 to 2003 to extend the follow-up. |
| Clinical endpoints | The aim of the study was to compare the effect of tamoxifen treatment vs no treatment (control) in relation to recurrence-free survival (RFS, primary outcome) and overall survival (OS, secondary outcome). RFS included local, regional, distant recurrences and breast cancer-specific death, but not contralateral breast cancer, as the primary event. |
| Variables examined or considered | Age, lymph node status, tumor size, grade (Nottingham Histological Grading), estrogen receptor status, Ki67 status, and pERK and SMAα status. Since HER2 has no predictive role in this series [2]. HER2 was not included in the multivariable model. |
| Rationale for sample size | The trial was planned to include at least 500 patients in a two-armed study aiming at a 15% difference in outcome between the arms, with 90% power and an alpha level of 5%. |

| **Methods (5)** | **Statistical analysis** |
|---|---|
| Statistical methods and variable selection procedure | Differences in distributions between the two arms, for clinical data and tumour characteristics, were evaluated by means Fisher's Exact, Pearson's Chi-square or Mann-Whitney *U* tests. All analyses were performed by the intention-to-treat rule. Results are not adjusted for multiple testing. RFS was estimated according to the Kaplan–Meier method. Cox univariable regression analyses (*P* values that were based on the Wald statistics) were used to compare survival in different subgroups. The Cox multivariable proportional hazards model was performed as described previously (including age, tumor size, lymph node status, grade, and Ki67 status) and because pERK correlated with levels of ERα, ERα was added to the model as well [3]. In addition the multivariable model included an interaction variable (tamoxifen +/- x pERK). All *P* values are based on a two-sided test. All calculations were made with Statistical Package for the Social Sciences (SPSS) version 15.0 (SPSS Inc., Chicago, IL). |
| Missing data | Patients with a missing value for one of the variables were excluded from the multivariable analysis. |
| Marker handling in analysis | For comparison with prognostic and molecular markers, the continuous |

| | |
|---|---|
| | values of pERK (and SMAα) were used. For other analyses (survival, Cox regression), pERK (and SMAα) were analyzed in a binary fashion. We considered tumours with scores 0-1 as having low pERK level (or low SMAα expression) and tumours with scores 2 and 3 as having high pERK level (or high SMAα expression). This cut-off resulted in two subgroups that were equally sized for pERK and skewed for SMAα with 3.5-fold more in the high group. |

| **Results (1)** | **Data** |
|---|---|
| Flow of patients | See Figure S5 for description of patients (including events) excluded for this translational study. |
| Characteristics | See Table 1 |

| **Results (2)** | **Analysis and presentation** |
|---|---|
| Relation to standard prognostic variables | See Table 1, Table S1 and Table S2 |
| Univariate analysis | See Figures 2a-d, Figures S2b-e, S3b-e, S4a. |
| Multivariate analysis | See Table 2 and Table S3. Estimated effects with CIs for marker and all other variables in the model. Variables were entered into the model in one single step (Enter model). |
| Additional analyses | Venn diagrams see Figures S2a, S3a and S4b. Proportions of low and high pERK or SMAα and resulting overlap (Figures S2a and S4b). Proportions of low and high stromal and tumour pERK and resulting overlap (Figures S3a). |

| **Discussion** | |
|---|---|
| Interpretation, limitations and implication | Our results, to our knowledge, are the first results from a randomized trail, to show that ERK phosphorylation levels in tumour-associated fibroblasts predict tamoxifen response. Our study has some limitations. Insufficient tumour material led to a loss of 45 of the 324 patients with an ERα-positive tumour. Further, patients were treated with tamoxifen for 2 years because the superiority of 5 years had not yet been established when this study was conducted. Finally, we evaluated homogeneity of results across the two centres of the trial and observed no evidence of differential tamoxifen benefit in the smaller centres, which contributed approximately 20% of the patients in our analysis. |


1. Svensson S, Jirstrom K, Ryden L, Roos G, Emdin S, et al. (2005) ERK phosphorylation is linked to VEGFR2 expression and Ets-2 phosphorylation in breast cancer and is associated with tamoxifen treatment resistance and small tumours with good prognosis. Oncogene 24: 4370-4379.
2. Ryden L, Jonsson PE, Chebil G, Dufmats M, Ferno M, et al. (2005) Two years of adjuvant tamoxifen in premenopausal patients with breast cancer: a randomised, controlled trial with long-term follow-up. Eur J Cancer 41: 256-264.
3. Allred DC, Harvey JM, Berardo M, Clark GM (1998) Prognostic and predictive factors in breast cancer by immunohistochemical analysis. Mod Pathol 11: 155-168.
4. Sargent DJ, Conley BA, Allegra C, Collette L (2005) Clinical trial designs for predictive marker validation in cancer treatment trials. J Clin Oncol 23: 2020-2027.
